# Multi-Line Fit Model for the Detection of Methane at *ν*_2_ + 2*ν*_3_ Band using Hollow-Core Photonic Bandgap Fibres

**DOI:** 10.3390/s90100490

**Published:** 2009-01-14

**Authors:** Ana M. Cubillas, Jose M. Lazaro, Olga M. Conde, Marco N. Petrovich, Jose M. Lopez-Higuera

**Affiliations:** 1 Photonics Engineering Group, University of Cantabria, Avda. de los Castros S/N, 39005 Santander, Spain; E-mails: josemiguel.lazaro@unican.es (J. M. L.); olga.conde@unican.es (O. M. C); miguel.lopezhiguera@unican.es (J. M. L.); 2 Optoelectronics Research Centre, University of Southampton, Southampton, SO17 1BJ, UK; E-mail mnp@orc.soton.ac.uk (M. N. P.)

**Keywords:** Gas sensing, Fibre optic sensors, Microstructure devices, Absorption spectroscopy, Hollow-Core Photonic Bandgap Fibres

## Abstract

Hollow-core photonic bandgap fibres (HC-PBFs) have emerged as a novel technology in the field of gas sensing. The long interaction pathlengths achievable with these fibres are especially advantageous for the detection of weakly absorbing gases. In this work, we demonstrate the good performance of a HC-PBF in the detection of the *ν*_2_ + 2*ν*_3_ band of methane, at 1.3 *μ*m. The Q-branch manifold, at 1331.55 nm, is targeted for concentration monitoring purposes. A computationally optimized multi-line model is used to fit the Q-branch. Using this model, a detection limit of 98 ppmv (parts per million by volume) is estimated.

## 1. Introduction

Gas sensing has become increasingly important as environmental awareness and industrial processes impose greater demands on measurement and monitoring systems [1, 2]. Spectroscopy-based techniques are well suited for monitoring gases as most of them have a characteristic absorption spectrum. Indeed, light passing through a gas is absorbed at specific wavelengths determined by its molecular structure. This absorption spectrum allows the identification of the gas and, at the same time, contains information about its concentration. Hence, spectroscopic-based gas-sensing systems are attractive for gas detection as they provide high spectral resolution, gas selectivity, precise identification of gas species and possibility of remote measurements [3]. Furthermore, the ability to use optical fibre waveguides as gas cells in spectroscopic-based sensors opens up the possibility to very long interaction lengths with the gas, thus (assuming the transmission loss is low) to very high sensitivity. Moreover, fibres offer additional advantages such as compact size, light weight, very small volume samples, possibility of distributed measurements and better integrability in optical systems. Until recently, systems based on conventional all-solid fibres showed a very poor performance [4]. In contrast, the advent of Hollow-Core Photonic Bandgap Fibres (HC-PBFs) has provided a more efficient platform to exploit the light-gas interaction [5].

HC-PBFs are a new class of optical fibres in which light is guided by virtue of a periodic array of micro-sized holes, i.e. a microstructure, running down the full fibre length. Such array of holes gives rise to an optical bandgap, i.e. an interval of wavelengths or frequency range where light cannot propagate through the microstructure. When an oversized air hole is introduced in the centre of the microstructure, a defect is created allowing the propagation of light. This central air hole forms the core of the fibre where light is trapped by the photonic bandgap determined by the cladding [5, 6].

HC-PBFs have unique properties that make them particularly suitable for gas sensing. When the hollow core of the fibre is filled with gas, very long interaction lengths between light and the gas confined in the core can be achieved, enabling high sensitivity measurements. Furthermore, HC-PBFs are also interesting for their possibilities of integrability in optical systems and compactness. For the aforementioned reasons, in the past few years gas-filled HC-PBFs have been widely investigated in applications such as gas detection [7, 8], high-resolution spectroscopy experiments [9, 10], wavelength locking [11] and nonlinear-optics [12].

The long pathlengths provided by HC-PBFs are particularly advantageous for monitoring weakly absorbing gases. Specifically, this work focuses on the detection of methane band *ν*_2_ + 2*ν*_3_, at 1.3 *μ*m. This region is of great interest as it is used as a telecommunication band. Therefore, it benefits from the fully-developed and low-cost telecommunication light sources and detectors available in this wavelength range. However, due to the weakly absorbing lines of this band, it is very difficult to detect with conventional gas cells. Traditionally, bulky gas cells have been needed to reach a good sensitivity [13]. We expect that the long pathlengths available with HC-PBFs would be able to compensate for the weak absorption lines of methane at 1.3 *μ*m-band and yield to a good sensitivity.

In a previous work, we investigated the potential of using a HC-PBF to detect methane [14]. The present work is intended to prove that it is also possible to measure low concentrations of methane at *ν*_2_ + 2*ν*_3_ weakly absorbing band using a HC-PBF as gas cell. The Q-branch, at 1331.55 nm, is targeted as it shows the strongest absorption within the 1.3 *μm* band. Furthermore, the detection limit of the system will be assessed by analyzing the spectral absorption of the Q-branch. In order to fit this manifold, a computationally optimized model is proposed. In a complementary work, we proved the good performance of a similar model in the fitting of a 6-transition manifold of methane [15]. However, this work tries to demonstrate that this model can also reduce the computational cost and considerably improve the detection limit of the system in a more complex analysis such as the Q-branch of methane at *ν*_2_ + 2*ν*_3_ band.

## 2. Theory of absorption spectroscopy

Absorption spectroscopy follows, in a wide set of circumstances, the Beer-Lambert law [1]. This law states that the transmission of light through a gas, *T*(*ν*), at a frequency *ν* [cm^−1^], is related to the properties of the gas species through which the light is traveling by the following expression:

(1) $$T(\nu )=\frac{I_{t}}{I_{o}}=\exp(-k_{\nu }L)$$

where *I_t_* and *I_o_* are the transmitted and input light intensities, respectively, *k_ν_* [cm^−1^] the spectral absorption coefficient and *L* [cm] the pathlength the light travels through the gas. The spectral absorption coefficient is expressed as:

(2) $$k_{\nu }=\sum_{i=1}^{N}S_{i}(T)\phi (\Delta \nu ,\nu )Px$$

In this case, *N* represents the number of transitions within a manifold that contribute to the absorption, *i* a specific transition, *P* [atm] the total pressure of the medium, *x* the mole fraction of the gas species (related to the gas concentration), *S_i_* (either in units of [cm^−2^atm^−1^] or [cm^−1^/(mol cm^−2^)]) the line strength for transition *i* at temperature *T* and *ϕ* [cm] is the normalized lineshape function of width Δ*ν*.

Furthermore, Equation 1 can be converted to spectral absorbance, *α*(*ν*) [AU], using:

(3) $$\alpha (\nu )=-\ln\left(\frac{I_{t}}{I_{o}}\right)=\sum_{i=1}^{N}S_{i}(T)\phi_{i}(\Delta \nu ,\nu )\text{P}xL$$

The lineshape function may be represented by a Gaussian profile (see Equation 4) when the effects of line broadening are due to thermal motion (Doppler broadening). The width of the lineshape is then described by the Doppler width, Δ*ν_D_* (FWHM).

(4) $$\phi_{D}(\Delta \nu_{D},\nu )=\frac{2}{\Delta \nu_{D}}{\left(\frac{\ln2}{\pi }\right)}^{1/2}\exp\left[-4\ln2{\left(\frac{\nu -\nu_{0}}{\Delta \nu_{D}}\right)}^{2}\right]$$

(5) $$\Delta \nu_{D}=7.1623\times 10^{-7}\nu_{0}{\left(\frac{T}{M}\right)}^{1/2}$$

where *M* is the molecular weight of the gas species.

Alternatively, collisional broadening, due to pressure, gives rise to a Lorentzian lineshape function (Equation 6), characterized by the collisional width parameter, Δ*ν_C_* (FWHM).

(6) $$\phi_{C}(\Delta \nu_{C},\nu )=\frac{2}{\pi }\frac{\Delta \nu_{C}}{{\left(\nu -\nu_{0}\right)}^{2}+{\left(\frac{\Delta \nu_{C}}{2}\right)}^{2}}$$

(7) $$\Delta \nu_{C}=P\sum_{B}x_{B}2\gamma_{A-B}$$

where A is the molecule whose lineshape is under study and B is the perturbing molecule. *γ_A_*_–_*_B_* is the collisional halfwidth per unit pressure of *A* molecule due to perturber *B.*

When both effects are significant, the resultant lineshape function is typically addressed using a Voigt profile [16]. Specifically, the lineshape can be described by a Gaussian function in low-pressure regimes. At high pressure, collisions of the molecules are dominant and the lineshape is described by a Lorentzian profile. At intermediate pressure, a Voigt profile is normally used.

## 3. Experimental setup

**Figure 1.**
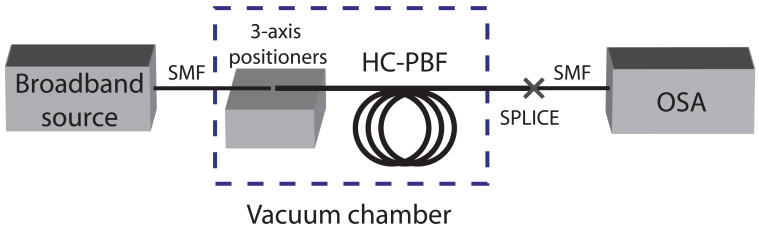
Experimental setup for methane detection experiments using a HC-PBF as gas cell.

Figure 1 shows an schematic of the arrangement used for methane detection experiments. The main difference with a conventional spectroscopic gas sensor is the substitution of the bulky conventional gas cell with a 5.6-m-long HC-PBF. It should be noted that, despite the length of the fibre, the system can be arranged in compact form as the fibre can be coiled up.

As can be seen in Figure 1, light from a broadband light source, (Agilent 83437A), was launched into a Single Mode fibre (SMF). To avoid reflections, the SMF was angle cleaved and coupled to the HC-PBF using 3-axis positioners. A gap was left between the ends of the fibres to allow the gas access into the core of the HC-PBF. The other end of the HC-PBF was spliced to a SMF pigtail using a similar procedure as described in [17]. The splice attenuation was measured to be 1 dB. The transmitted power through the HC-PBF was measured using an Optical Spectrum Analyzer (OSA), (Agilent 86142A). The HC-PBF, with its open end, was placed inside an airtight chamber, as illustrated in Figure 1. Finally, a pump was used to evacuate the air from the chamber and a pressure gauge monitored the vacuum conditions inside.

The HC-PBF used in the experiments was especially designed and manufactured by the Optoelectronics Research Centre at Southampton. The fibre was fabricated using a two-step stack-and-draw process [6]. In this case, 7 capillaries from the centre of the microstructure were omitted to form the hollow-core where gas can be contained. A high-resolution scanning electron microscope (SEM) image of the cross-section of the HC-PBF is shown in 2. The structural parameters of the fibre were: outer diameter of 188 *μ*m, cladding pitch of 2.9 *μ*m, air filling factor of the cladding of 95% and core diameter of 10.3 *μ*m.

**Figure 3.**
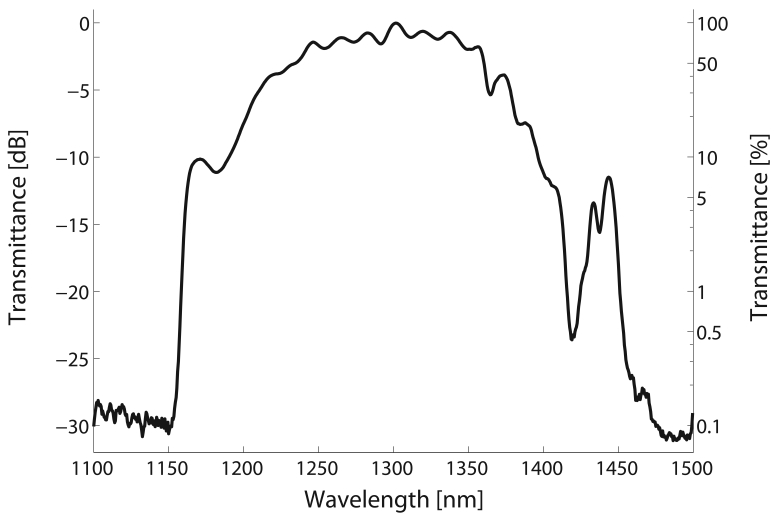
Normalized spectral transmission of the 5.6-m-long HC-PBF used in the experiments.

Moreover, the HC-PBF has a bandgap guidance in the region 1150-1450 nm, with an estimated minimum loss of 100 dB/km (see Figure 3). The fibre shows low attenuation in the 1300-1350 nm wavelength range. According to the spectroscopic database HITRAN [18], the bandgap of the HC-PBF is particularly suitable for measuring the *ν*_2_ + 2*ν*_3_ band of methane, located around 1310-1345 nm.

## 4. Experimental results and Discussion

### 4.1. Selection of spectral lines

**Figure 4.**
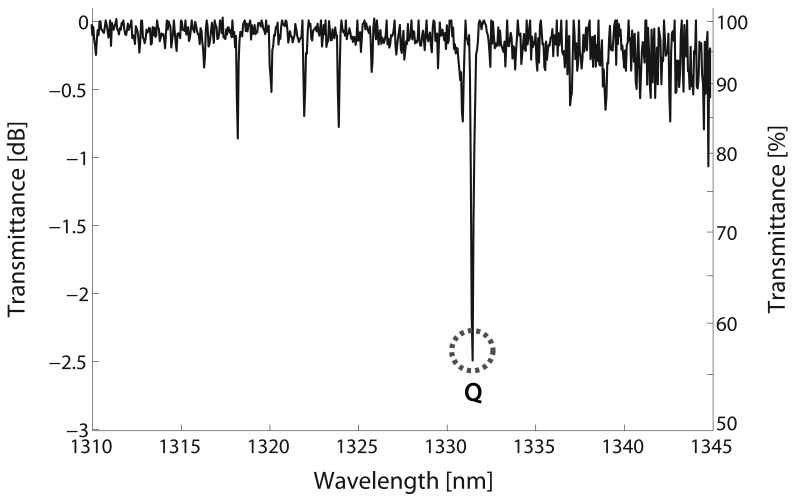
Transmission spectrum of the *ν*_2_ + 2*ν*_3_ band of methane, at 1.3 *μ*m, measured at relative pressure of 1 bar, room temperature and 18750 ppmv calibrated concentration of methane in air.

The selection of a suitable absorption line is one of the foremost steps in the design of a gas sensor. For that purpose, methane transmission spectrum at *ν*_2_ + 2*ν*_3_ band, at 1.3 *μ*m, was measured with the setup described in Figure 1. In this case, the vacuum chamber was filled with a calibrated mixture of 18750 ppmv (parts per million by volume) methane in air at room temperature and a relative pressure of 1 bar. In order to take into account only the absorption due to the gas, the acquired spectrum was normalized to the transmission spectrum obtained when the chamber was set to vacuum. The resulting spectrum can be seen in Figure 4. In this case, the transmission spectrum for methane was recorded from 1310 to 1345 nm with a resolution of 0.05 nm.

For gas detection purposes, a strong absorption line free from interference by other gas species is desired to guarantee a high Signal-to-Noise Ratio (SNR). As can be seen, the Q-branch peak, circled in Figure 4, is considerably higher than the other peaks in the spectrum. For that reason, it was selected for methane sensing experiments.

### 4.2. Filling dynamics of the HC-PBF

Using the arrangement of Figure 1, the gas flow dynamics inside the HC-PBF was also investigated. This is a critical parameter in the design of a fibre-based gas sensing system as it highly influences the response time of the sensor. Although the vacuum chamber can be filled with gas in a few seconds, the time needed for the gas to diffuse along the fibre is strongly dependent on the gas species, the pressure, the core diameter and the length of the fibre [7, 19].

**Figure 5.**
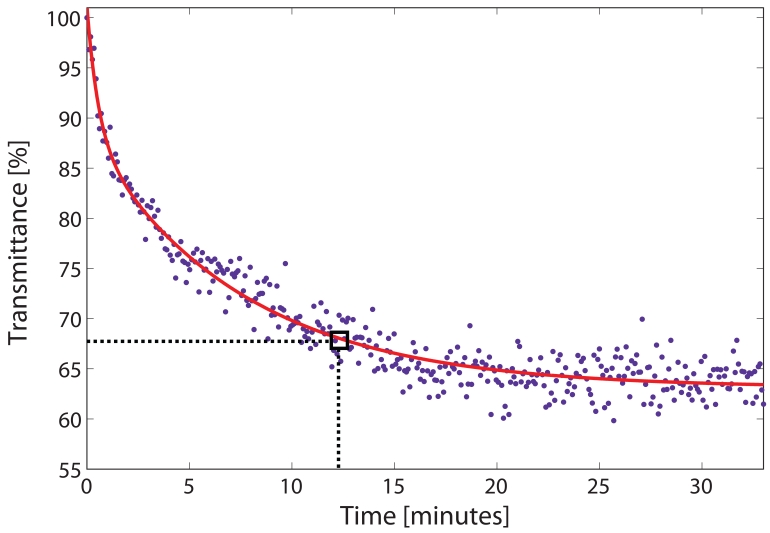
Normalized transmittance as a function of time measured at the center of the Q-branch, at 1331.55 nm. Dots represent the experimental data and solid red line, the double exponential fit. The filling time is boxed.

To evaluate the filling process, the power of the light transmitted through the HC-PBF was monitored at the centre of the Q-branch (1331.55 nm) when the chamber was filled with gas at a relative pressure of 1 bar. The light transmission profile as a function of time is depicted in Figure 5. The experimental fit of the data was empirically determined using a double exponential function (see solid red line in Figure 5). We defined the filling time as the time needed for the gas to reach a transmittance value 5% higher than that of the steady value. In this case, around 12 minutes were required for the gas to diffuse inside the fibre (see box in Figure 5). It is clear that this diffusion time might limit the use of a gas sensor based on a HC-PBF in fast-response applications. Nevertheless, this time can be considerably shortened by replacing the one piece HC-PBF by shorter segments of HC-PBF separated by small gaps [20, 21].

### 4.3. Data analysis and detection limit calculation

**Figure 6.**
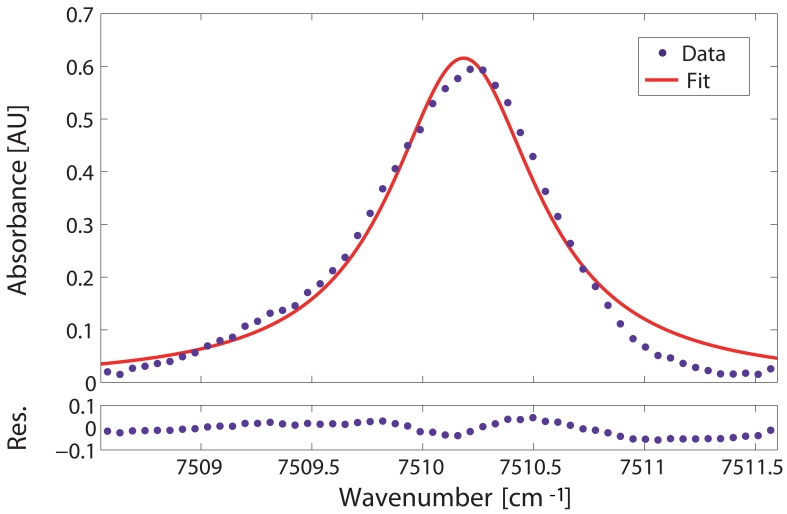
Upper panel: Single Lorentzian fit (solid curve) and experimental absorbance data (dots) for the Q-branch at room temperature, relative pressure of 1 bar and methane concentration of 18750 ppmv. Lower panel: Residuals of the fit.

The detection limit of the system was assessed by analyzing the absorbance of the selected gas line. Therefore, the absorbance of the Q-branch was calculated from the input and transmitted intensities (see Equation 3) measured when the vacuum chamber was filled with a calibrated methane-air mixture of 18750 ppmv methane at room temperature and 1 bar of relative pressure. Specifically, the transmitted intensity was measured directly from the fibre whereas the input intensity was determined by fitting a low-order polynomial to the regions of the transmitted sprectrum where there was no absorption. Additionally, the absorption profile was corrected with the transmission spectrum when the gas chamber was in vacuum conditions. In this way, the result was corrected for the transmission loss of the HC-PBF spectrum and the other coupling losses from the setup. The experimental absorbance values of the Q-branch, calculated as described above, are shown as dots in the upper panels of Figures 6 and 8.

Gas absorption lines are usually fit using a single lineshape function. At the pressure and temperature conditions of our experiment, the effect of thermal broadening was negligible and only collisional broadening was considered in this study (Δ*ν_C_* ≫ Δ*ν_D_*). Thus, the lineshape function of the absorption lines was represented with a Lorentzian function (see Equation 6) [22]. As an initial approach, the absorption profile of the whole Q-branch was fitted via a Levenberg-Marquardt algorithm to a single Lorentzian line using Equation 3. In this case, the free parameters of the fit were the linestrength *S_i_*, the collisional linewidth Δ*ν_Ci_* and the line position *ν*_0_*_i_*. The result can be seen as a solid line in the upper panel of Figure 6. The residuals, calculated as the difference between the experimental data and the best-fit profile, are shown in the lower panel of Figure 6.

From the fit, the signal-to-noise ratio as well as the detection limit of the system can be obtained. The SNR was calculated as the ratio of the absorption peak amplitude to the standard deviation of the residuals from the fit. In this case, a SNR of 21.5 (i.e. 13.3 dB) was estimated. This yields to a concentration detection limit, *x_min_*, (by extrapolation to SNR=1) of 870 ppmv. The minimum detectable absorbance (MDA) of the system can also be calculated with the following expression:

(8) $$\text{MDA}=\sum_{i=1}^{N}S_{i}(T)\phi_{i}(\nu_{0})Px_{\text{min}}L$$

In this case, a MDA of 2.833×10^−2^ AU was predicted.

The results here obtained show a very poor performance in terms of sensitivity for an absorption spectroscopic-based sensor. This is mainly due to the inaccuracy of the fit performed, which used a single Lorentzian line to fit the Q-branch manifold. Indeed, there clearly is not a good agreement between the experimental data and the curve fit in Figure 6. The Q-branch at *ν*_2_ + 2*ν*_3_ band is composed not by a single but multiple, closely spaced energy transitions, as deduced from HITRAN database [18]. Specifically, in the wavelength range scanned, there are 83 listed transitions, some of them of considerable importance (due to their strength) in the curve fitting of the Q-branch. Furthermore, under our experimental conditions (room temperature, 1 bar of relative pressure), these transitions are strongly broadened and overlapped into a broad spectral line, which greatly complicates the spectral analysis of the Q-branch. However, the much stronger absorption of the Q-branch as compared to the other lines in the band justifies its choice in our detection scheme. It becomes, therefore, necessary to find a model to accurately fit the manifold at as low computational cost as possible.

As stated before, in order to fit an absorption line to Equation 3, three free parameters per transition need to be determined. In the case of the Q-branch manifold, this yields to the calculation of over 200 unknown parameters, which may result in a very high computational cost. We applied the method described in reference [23] for our calculations. This method uses some simplifications in Equation 3, which reduce the number of parameters and hence, the computational cost, while providing results that are in good agreement with the HITRAN database [18]. More in detail, the collisional broadening was assumed equal for all the transitions in the manifold, i.e. Δ*ν_C_*. Furthermore, at room temperature the individual linestrengths of each transition, *S_i_*, are expressed in terms of a total linestrength of the manifold, *S_tot_*, and the relative contribution of each transition to the total linestrength, *ω_i_*. Finally, the central frequency of each transition, *ν*_0_*_i_*, is represented using an arbitrary central frequency, *ν*_0_*_R_*, and the wavenumber spacing of each transition to that, *δν_i_*. Both *ω_i_* and *δν_i_* values are known and derived from HITRAN database [18]. These simplifications yield to a just 3-parameter model (i.e. *S_tot_, ν*_0_*_R_*, Δ*ν_C_*) to handle the overall transitions that comprise the Q-branch manifold:

(9) $$\alpha (\nu )=PL_{x}S_{\text{tot}}\sum_{i=1}^{N}\omega_{i}\phi_{i}(\Delta \nu_{C},\nu -\nu_{0R}-\delta \nu_{i})$$

Now, the key point is how many transitions are necessary to consider in Equation 9 in order to accurately model the Q-branch while retaining the best trade-off in terms of computational cost. In order to test the efficiency of the model proposed, the detection limits for eight cases considering different number of transitions in the model of Equation 9 were evaluated. Thus, for each case, we selected only the transitions above an arbitrary linestrength (as listed in HITRAN database [18]). Specifically, the minimum linestrengths were 16×10^−23^, 14×10^−23^, 10×10^−23^, 8×10^−23^, 6×10^−23^, 4×10^−23^, 2×10^−23^ and 10×10^−24^ cm^−1^/(mol cm^−2^) (i.e. 1, 2, 3, 4, 10, 13, 26 and 36 transitions in the model, respectively). For the previous cases, the Q-branch absorbance measurements were non-linear least-squared fitted with a Levenberg-Marquardt algorithm using Equation 9. Both the detection limit and the computational time required for each fit were computed and the results are shown in Table 1.

**Figure 7.**
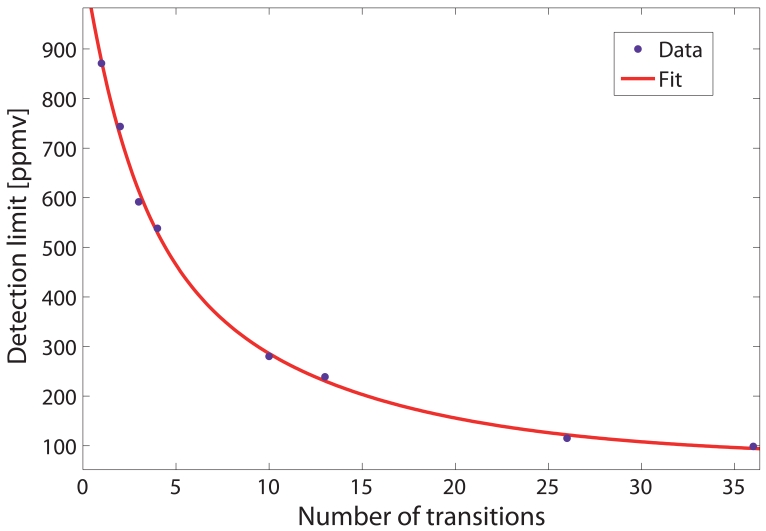
Detection limit of the system as a function of the number of transitions considered in Equation 9. The experimental values are shown as dots and the double exponential fit to the data as a solid red line.

Furthermore, the dependence of the detection limit as a function of the number of transitions assumed in the model is shown in Figure 7. The experimental values are shown as dots and the double exponential fit to the data as a solid red line. It can be seen that the fact of using more number of transitions in the fit considerably improves the detection limit of the system.

**Figure 8.**
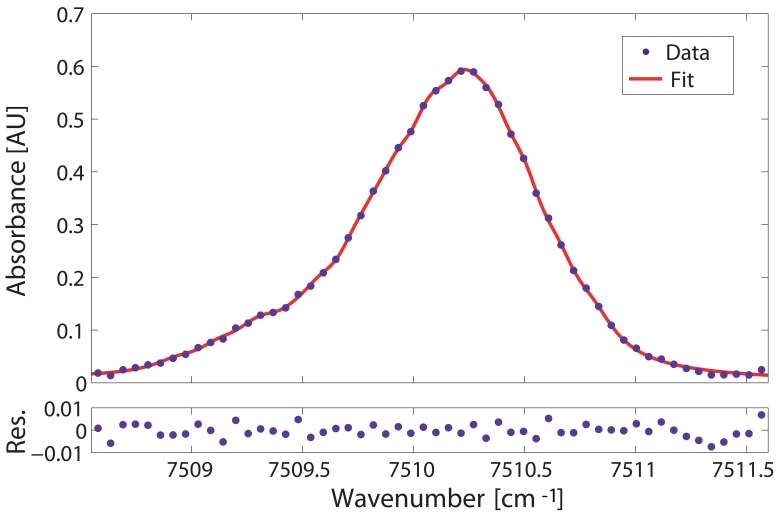
Upper panel: 36-transition Lorentzian fit (solid curve) and experimental absorbance data (dots) for the Q-branch at room temperature, relative pressure of 1 bar and methane concentration of 18750 ppmv. The detection limit is 98 ppmv. Lower panel: Residuals of the fit.

Figure 8 clearly demonstrates a significant improvement of the fitting of the Q-branch manifold when 36 transitions are used in the model of Equation 9 (solid red line). The plot shows an excellent agreement between experimental and calculated lineshape where residual values are greatly minimized. A SNR of 191 (i.e. 22.8 dB) was obtained, which yields to a concentration detection limit of 98 ppmv. This result improves the previous detection limit by a factor of 10 without requiring a significant increase in computational cost. Finally, a MDA of 3.1053×10^−3^ AU was predicted for the system configuration, in agreement with previously reported MDAs for absorption spectroscopic systems [24].

## 5. Conclusions

A multi-line fit model for the detection of methane at *ν*_2_ + 2*ν*_3_ band using a Hollow-Core Photonic Bandgap Fibre is proposed and demonstrated in this paper. The unique properties of HC-PBFs make possible the realization of a compact, sensitive and highly integrable gas sensor. In this work, the *ν*_2_ + 2*ν*_3_ band of methane, at 1.3 *μ*m, was targeted as it benefits from the fully-developed and low-cost sources and detectors available at the second telecommunication window. For that purpose, a HC-PBF specially suited for the detection of the *ν*_2_ + 2*ν*_3_ band was designed and fabricated. The Q-branch manifold, at 1331.55 nm, was selected for concentration measurements because it showed the best SNR in the band. Due to the great complexity of the spectral analysis of the Q-branch, we made use of a computationally optimized multi-line model to fit the manifold. As a result, we estimated a sensitivity of 98 ppmv for the system configuration. This yields to an improvement by a factor of 10 over the detection limit of a single-line fit model, without significantly increasing the computational cost. Furthermore, the gas flow dynamic process inside the HC-PBF was also investigated. Although the long diffusion time of the gas inside the fibre is still an issue, we are currently conducting research to reduce this time. Finally, the system here proposed can be extended to the detection of other gases in the near-infrared region.

## Figures and Tables

**Figure 2.**
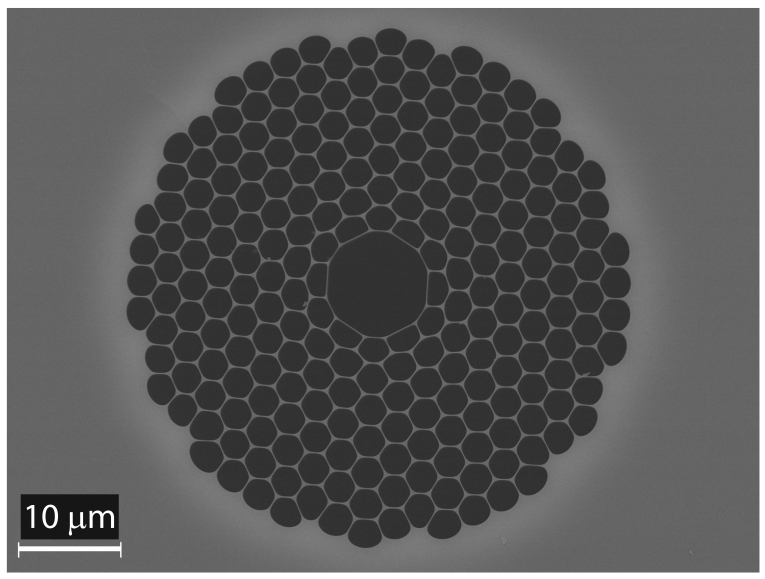
SEM image of the cross-section of the HC-PBF used in the experiments.

**Table 1.** Detection limit and computational time required according to the number of transitions considered.

Minimum *S_i_* [cm^−1^/(mol cm^−2^)]	Number of transitions	Detection limit [ppmv]	Computational time [s]
16×10^−23^	1	870	0.113
14×10^−23^	2	744	0.121
10×10^−23^	3	592	0.131
8×10^−23^	4	538	0.141
6×10^−23^	10	280	0.177
4×10^−23^	13	239	0.205
2×10^−23^	26	115	0.347
10×10^−24^	36	98	0.431
